# Clinical Outcomes of Arthroscopic Notchplasty and Partial Resection for Mucoid Degeneration of the Anterior Cruciate Ligament

**DOI:** 10.3390/jcm10020315

**Published:** 2021-01-16

**Authors:** Joong Won Lee, Jung Tae Ahn, Hyun Gon Gwak, Sang Hak Lee

**Affiliations:** 1 Department of Orthopaedic Surgery, Kyung Hee University Hospital at Gangdong, Seoul 02447, Korea; aliense@hanmail.net (J.W.L.); jtahnos@gmail.com (J.T.A.); hyun8725@naver.com (H.G.G.); 2 Department of Orthopaedic Surgery, Kyung Hee University School of Medicine, Seoul 02447, Korea

**Keywords:** mucoid degeneration, anterior cruciate ligament, notchplasty, ganglion cyst, posterior septal portal

## Abstract

Background: Mucoid degeneration of the anterior cruciate ligament (MD-ACL) is a chronic degenerative process involving a hypertrophied ACL, which may lead to notch impingement syndrome. As a treatment method, there is consensus regarding arthroscopic resection for MD-ACL resulting in good clinical outcomes; however, additional notchplasty remains controversial. The purpose of this study was to investigate clinical outcomes after arthroscopic partial resection of the ACL and additional notchplasty performed to minimize volume reduction of the ACL. Study Design: Level IIIb retrospective cohort study. Methods: Of 1810 individuals who underwent knee arthroscopic surgery performed by the same surgeon between July 2011 and October 2020, 52 were included, while 10 were excluded due to a follow-up period of <1 year. Clinical data including pain location, terminal flexion or extension pain, range of motion (ROM), Lysholm knee score, and Hospital for Special Surgery (HSS) knee score were assessed pre- and postoperatively. Additionally, according to the resected volume of the ACL, patients were classified into two groups: <25% (Group 1), and 25–50% (Group 2). Clinical outcomes were compared between the two groups. Results: There were 17 (40.5%) men and 25 (59.5%) women with a mean age of 53.9 years (range, 16–81 years) at the time of surgery. The mean duration of symptoms before surgery was 14.4 months (range, 3–66 months). Arthroscopic partial resection of the MD-ACL was performed in all patients, and concomitant notchplasty was performed in 36 (81.8%). All clinical scores improved postoperatively, and were statistically significant (*p* < 0.01). However, there was no significant difference in clinical outcomes between groups 1 and 2 classified according to the resected ACL volume. Recurrence of MD-ACL was recorded in only one patient, 11 months after arthroscopic treatment. No patients underwent ACL reconstruction because of symptoms of anterior instability. Conclusion: Arthroscopic partial resection of the ACL and concomitant notchplasty yielded satisfactory outcomes for the treatment of MD-ACL. Notchplasty may be an alternative procedure to avoid total ACL resection and postoperative instability.

## 1. Introduction

Mucoid degeneration of the anterior cruciate ligament (MD-ACL) was first characterized by Kumar et al. in 1999 [1]. It is a chronic degenerative change of a bulky ACL that may lead to notch impingement syndrome in the absence of instability symptoms and significant trauma history [1,2,3,4]. Symptoms include insidious onset of posterior knee pain and limited range of motion (ROM) [4]. Magnetic resonance imaging (MRI) is the gold standard modality for diagnosis [5], and demonstrates a highly increased signal of a bulky ACL on T2-weighted and proton density-weighted images, which occupies almost the entire intercondylar notch and has been defined as the “celery stalk” sign [4,5,6]. Arthroscopy is an additional diagnostic tool which is used for treatment. In arthroscopy, ACL fibers are exposed because they have no synovial coverage, and a yellow or brown substance is apparent through the anterior fibers of the ACL or by probing between them [4,5]. The prevalence of MD-ACL on MRI has been reported to range from 0.43% to 5.3%. [1,7,8] Despite the relatively high prevalence of MD-ACL, its pathogenesis remains unclear. [4,9] Nevertheless, several studies have addressed the pathogenesis of MD-ACL and have proposed traumatic, synovial, and degenerative theories [5,9,10,11]. Trauma-based theories have addressed the relationship between the anatomy of the intercondylar notch and MD-ACL [6], and the impingement of a hypertrophied ACL on a narrow intercondylar notch has been proposed as one possible cause [6,12,13]. In this regard, previous reports have suggested arthroscopic resection of a hypertrophied ACL as a treatment method, including a consensus on arthroscopic resection for MD-ACL resulting in good clinical outcomes, including pain relief and ROM recovery [4,6,10,14,15]. However, there is a lack of literature that has focused on the resected volume of the ACL, nor is there a consensus regarding how much of the ACL should be removed. Furthermore, whether to undergo an additional notchplasty still remains controversial [4,6,14,15,16]. We hypothesized that additional notchplasty could be an alternative procedure to avoid total resection of the ACL and postoperative instability [5,13]. The purpose of this study, therefore, was to investigate clinical outcomes after arthroscopic partial resection of the ACL and additional notchplasty performed to minimize volume reduction of the ACL.

## 2. Materials and Methods

### 2.1. Clinical Data of Patients

**Figure 1:**
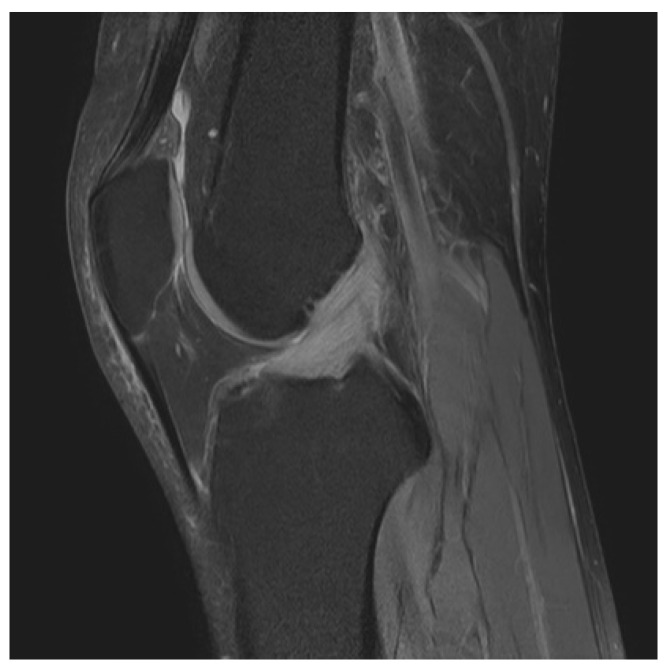
Preoperative proton density-weighted sagittal view of magnetic resonance imaging (MRI) shows overall high signal intensity and increased diameter of the anterior cruciate ligament (ACL) without discontinuity.

**Figure 2:**
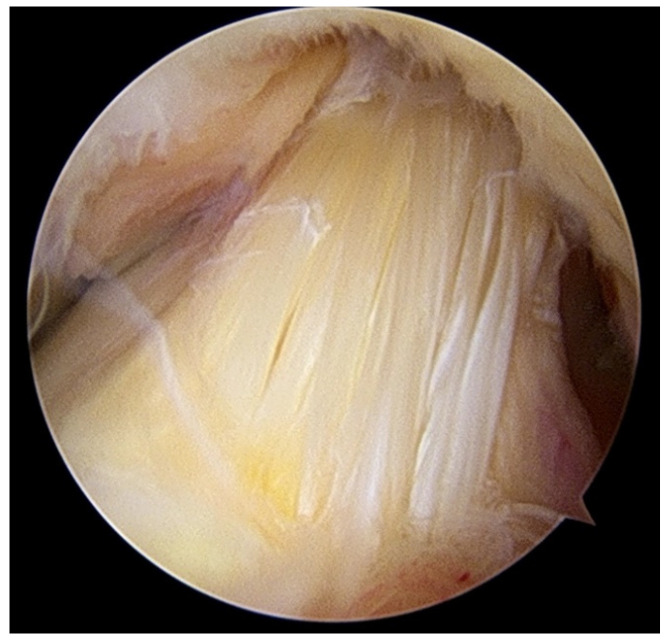
Arthroscopic findings through anterolateral portal of the left knee show absence of synovial lining with longitudinal separation of the anterior cruciate ligament (ACL) bundles.

Between July 2011 and October 2020, a total of 1810 patients underwent knee arthroscopic surgery performed by the same surgeon. MD-ACL was diagnosed according to clinical, MRI, and arthroscopic findings in all patients. The inclusion criteria for MD-ACL on MRI findings were as follows: overall high signal intensity of the ACL in T1- and T2-weighted images without discontinuity, increased ACL diameter, and poor visualization with thickened ACL fibers (Figure 1). The inclusion criteria for MD-ACL on arthroscopic findings were intact ACL fibers without evidence of tears, and partial or absent synovial lining with longitudinal separation of the ACL bundles [5] (Figure 2). Fifty-two patients were included based on these criteria; however, 10 were excluded due to a follow-up period of <1 year. Patients underwent knee arthroscopic surgery only after a minimum of 3 months of conservative management consisting of oral analgesia and strengthening exercises. ACL diameter and combined intra-articular pathologies were evaluated on preoperative MRI, and degenerative changes according to the Kellgren–Lawrence grade system were assessed using radiographs. Clinical data, including pain location, terminal flexion or extension pain, ROM, Lysholm knee score, Hospital for Special Surgery (HSS) knee score, and ligament instability, were collected pre- and postoperatively. The latest scores were used in the analysis. We measured the resected volume of the ACL pre- and postoperatively using the length of the probe as a reference. Additionally, patients were classified into two groups according to the resected volume of ACL: <25% (group 1); and 25–50% (group 2). Clinical outcomes were compared between the two groups.

### 2.2. Surgical Procedure

**Figure 3:**
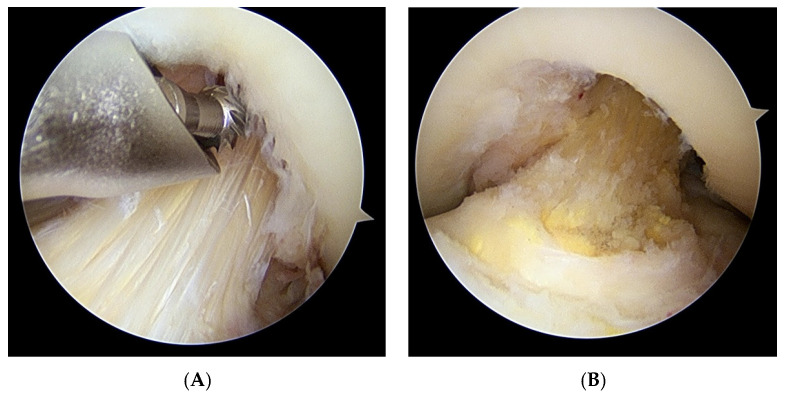
(**A**). Arthroscopic findings through the anterolateral portal of the left knee show a narrow intercondylar notch and hypertrophied anterior cruciate ligament (ACL. Notchplasty was performed first by using a motorized burr. (**B**). The hypertrophied ACL was then resected to <50% of the volume to achieve impingement-free motion between the ACL and intercondylar notch.

All patients underwent arthroscopy using the same arthroscopic technique with a tourniquet. Routine arthroscopic knee examination was performed through the anteromedial and anterolateral portals. During arthroscopy, all compartments were examined to evaluate ligaments, menisci, and cartilage lesions. Arthroscopic treatment consisted of partial resection of the ACL and notchplasty [5,6,14]. The goal was to preserve as many fibers of the ACL as possible, and to remove conflict with the notch during flexion and extension motion [16]. In knees with obvious impingement of the hypertrophied ACL on a narrow notch, notchplasty was performed first using a motorized burr or curved osteotome to remove the osteophyte. The hypertrophied ACL was then resected to <50% of the volume to achieve impingement-free motion between the notch and the ACL (Figure 3). In knees without intercondylar notch narrowing, partial resection of the bulky ACL was performed first, and additional notchplasty was performed if needed. If necessary, the additional posterior septal portal was established to remove all visible impinging structures, including ganglion cysts in the posterior compartment. Postoperatively, no brace or immobilizer was used. All patients started early mobilization with quadriceps strengthening exercises and allowed full weight-bearing ambulation according to pain tolerance.

### 2.3. Statistical Analysis

The results were analyzed using SPSS version 21.0 (IBM Corporation, Armonk, NY, USA) and G*Power version 3.1.5 (Faul, Erdfelder, Buchner, and Lang, Germany). Comparisons between preoperative and final follow-up data, such as clinical scores and range, were performed using a paired t-test and Wilcoxon signed-rank test. Clinical outcomes between group 1 (i.e., <25% volume of the ACL resected) and group 2 (i.e., 25% to 50% volume of the ACL resected) were analyzed using the independent *t*-test or Mann–Whitney test. Differences of *p* < 0.05 were statistically significant. Post hoc power analysis was performed with an α error of 5% and a β error of 20% to detect differences in the mean values between preoperative and postoperative values. The power of all preoperative and postoperative results was over 0.95, which was very robust. Moreover, the post hoc power was analyzed using the Chi-square goodness-of-fit test with an α error of 5% and a β error of 20% to detect differences in the proportion of notchplasty between the groups. Based on this calculation, the power was robust (>0.97) with an effect size of 0.6285393.

## 3. Results

The present study enrolled 42 patients who were followed up for a mean of 14.4 months (range, 12–106 months) after surgery. There were 17 (40.5%) men and 25 (59.5%) women, with a mean age of 53.9 years (range, 16–81 years) at the time of surgery. The mean duration of symptoms before surgery was 14.4 months (range, 3–66 months). The clinical characteristics of the 42 patients included in the present study are summarized in Table 1.

The chief complaint of all patients was insidious onset of posterior knee pain (84.1%) exacerbated by flexion or extension without a significant history of trauma.

On MRI, the mean (± standard deviation) sagittal diameter of the ACL was 8.99 ± 0.38 mm (range, 5.21 to 15.16 mm). Based on MRI and arthroscopic findings, ganglion cysts of the ACL and meniscal tears were the most commonly found in 28 (66.7%) patients, and chondral lesions of International Cartilage Repair Society (ICRS) grade ≥ 2 in 23 (54.8%) patients (Table 2). Arthroscopic partial resection of the MD-ACL was performed in all patients, and concomitant notchplasty in 36 (81.8%) patients (Table 3). Other combined intra-articular pathologies were also treated arthroscopically. The combined ganglion cysts or popliteal cysts were resected for all patients. Meniscal tears were treated with partial meniscectomy in 24 (57.1%) patients or repair in 4 (9.5%) patients. In addition, 16 (38.1%) patients with ICRS grade ≥2 cartilage lesions were treated with chondroplasty. For debridement of the posterior compartment, a posterior septal portal was established in 20 (47.6%) patients, as described by Ahn et al. [17].

Pre- and postoperative clinical results, including Lysholm knee scores, Hospital for Special Surgery knee scores, degree of flexion and extension deficit, are summarized in Table 4. All clinical scores improved postoperatively and were statistically significant (*p* < 0.001). However, there was no significant difference in clinical outcomes between the groups classified according to resected ACL volumes (i.e., group 1 and 2) (Table 5). Recurrence of MD-ACL was recorded in only one patient 11 months after arthroscopic treatment. No patients underwent ACL reconstruction prompted by symptoms of anterior instability.

## 4. Discussion

The present study included 42 patients with MD-ACL who complained of insidious onset of knee pain (especially posterior knee pain) and limitation of ROM without a history of trauma. We report excellent clinical results after arthroscopic partial resection of MD-ACL and additional notchplasty. One of the 42 patients (2.4%), however, experienced recurrence of a ganglion cystic lesion around the cruciate ligament, resulting in pain and limitation of ROM 11 months after arthroscopic treatment. This patient exhibited severe synovitis on arthroscopic examination at the time of surgery. The reason for recurrence was not clear; however, this patient was later diagnosed with rheumatoid arthritis, and chronic synovitis resulting from rheumatoid arthritis may be a possible explanation. Although the patient is currently undergoing conservative treatment, including oral rheumatoid medication, pain and swelling persist. Patients were also classified into two groups according to the resected volume of the ACL (i.e., group 1 and 2) and compared; however, there was no significant difference in clinical outcomes between the two groups.

Although the disease entity of MD-ACL remains unclear, studies in the literature have reported that the prevalence of MD on MRI is 0.43% to 5.3% [3,7,8]. Several others have investigated the pathogenesis of MD-ACL and have proposed traumatic, synovial, and degenerative theories [5,9,10,11,18]. Among these, impingement of the hypertrophied ACL on the narrow intercondylar notch has been suggested as one of the possible causes [6,12,13]. Therefore, as a treatment method, there is consensus regarding arthroscopic resection of the hypertrophied ACL; however, controversy persists regarding the extent of ACL removal and whether concomitant notchplasty should be performed. Moreover, the issue of instability after arthroscopic resection of the ACL is debatable. At first, Kumar et al. suggested that total resection of the affected ACL is a safe treatment method that does not result in anterior instability [1]. Subsequently, several studies have reported satisfactory clinical results after arthroscopic debridement of the ACL and additional notchplasty [4,6,14]. They suggested that arthroscopic debridement of the hypertrophied ACL and additional notchplasty should be performed when an impingement exists [3].

However, Lintz et al. treated their patients with partial resection of the ACL in 12 (41%) knees, total resection in 17 (59%), and additional notchplasty in only 2 (7%) [5]. They found that 28 of 29 (97%) knees exhibited a positive Lachman test, and the average of differential laxity according to the TelosTM device (Telos Medical, Austin and Associates, Millersville, MD, USA) was 8.3 mm (range, 5 to 13 mm). Anterior instability can be seen in ACL-deficient knees, and >5 mm of anterior tibial translation is generally considered as a cut off value for ACL reconstruction [19,20]. However, two patients underwent subsequent ACL reconstruction. Ventura et al. treated their patients with partial resection of the ACL in 8 (32%) knees and total resection in 17 (68%) without notchplasty [15]. They reported a negative pivot shift test and a positive Lachman test for all patients, and reconstruction of the ACL was performed in one young patient. Cha et al. reported that anterior translation >5 mm was found in seven (10%) patients postoperatively, four of whom complained of anterior instability [5,21]. These results appear to have been affected by the extent of ACL debridement. Kim et al. reported that none of their patients exhibited anterior instability [14]. Although they did not assess the resected volume of ACL, they performed notchplasty first in knees with notch narrowing, followed by debridement of the bulky ACL. Accordingly, we first decompressed the lateral wall by using a motorized burr while performing a flexion–extension maneuver when an obvious impingement was observed. In our cohort, notchplasty was performed in 66% of patients in group 1 and in 96% of those in group 2 (Table 5). This means that notchplasty was performed more frequently in patients with a narrow notch to reduce resected volume. In this regard, the resected volume of the ACL could have been reduced, and none of our patients underwent subsequent ACL reconstruction prompted by complaints of symptoms of anterior instability.

According to MRI and arthroscopic findings in our cohort, ganglion cysts related to the ACL were the most commonly found in 28 (66.7%) patients. In the literature, the incidence of ganglion cysts related to the ACL has been reported to be as high as 1.1% in arthroscopy, and 0.44% on MRI. [5,22,23,24]. Many of these ganglion cysts were found incidentally without symptoms and often coexisted with MD-ACL. Although the pathogenesis of ACL ganglion cysts remains controversial [23,25,26], in our opinion, there are similarities to those of MD-ACL, including trauma theories. Krudwig et al. [9] reported that symptoms, such as pain and limited ROM, were found in only 11% of their cases, and that these symptoms were related to the size and location of the ganglion cysts [25]. Parish et al. treated 15 patients with arthroscopic debridement and reported good clinical outcomes, including pain relief and recovery of ROM, without recurrence [23]. Mao et al. also reported good clinical outcomes without recurrence after arthroscopic debridement of the ganglion cyst in 11 patients [25]. However, ganglion cysts that extend to the posterior compartment are barely visible through anteromedial or anterolateral portals [27]. In a previous report, Tsai et al. suggested that using a posterior septal portal may be a good treatment option in patients with posterior ganglion cysts [27]. In our cohort, for excellent visualization and debridement of the posterior aspect of the ACL or ganglion cysts that extend to the posterior compartment, we performed arthroscopy using an additional posterior septal portal in 20 patients and demonstrated excellent clinical outcomes without recurrence [17].

The present study had a few limitations, the first of which was its retrospective design, and that postoperative anterior laxity was not evaluated using stress radiographs. Although the degree of anterior laxity was not measured, none of our patients underwent ACL reconstruction prompted by complaints of symptoms of anterior instability. Second, histological biopsy was not performed. Because MD-ACL exhibits characteristics similar to partial tears of the ACL, misdiagnosis was possible. However, we performed arthroscopic assessment in all patients, and the diagnosis of MD-ACL was based on clinical, MRI, and arthroscopic findings. Third, there was no control group that did not undergo notchplasty with which clinical results could have been compared. Furthermore, a relatively small number of included patients make it difficult to draw valid conclusions, although a power of more than 0.8 was achieved in some variables.

## 5. Conclusions

Arthroscopic partial resection of the ACL and concomitant notchplasty yielded satisfactory results for treating MD-ACL. Notchplasty could be an alternative procedure to avoid total ACL resection and postoperative instability.

## Figures and Tables

**Table 1:** Clinical findings of mucoid degeneration of the anterior cruciate ligament (MD-ACL) (*n* = 42).

	Number of Patients (%)
Posterior knee pain Pain on terminal flexion Pain on terminal extension	37 (84.1%) 38 (86.4%) 34 (77.3%)
Limitation of range of motion Flexion deficit Extension deficit	21 (50%) 20 (47.6%)

**Table 2:** Combined intra-articular pathologies with mucoid degeneration of the anterior cruciate ligament (MD-ACL) (*n* = 42).

Pathology	No. of Patients (%)
Combined ACL ganglion cyst	28 (66.7%)
Meniscal tears Medial Lateral Medial and Lateral Chondral lesion ICRS grade ≥2	28 (66.7%) 14 (33.3%) 4 (9.5%) 10 (23.8%) 23 (54.8%)
Medial condyle Lateral condyle Medial and Lateral condyle	10 (23.8%) 1 (2.3%) 12 (28.6%)
Patellofemoral chondral lesion Popliteal cyst	20 (47.6%) 5 (11.9%)

ICRS = International Cartilage Repair Society.

**Table 3:** Arthroscopic treatments of mucoid degeneration of the anterior cruciate ligament (MD-ACL) (*n* = 42).

	Number of Patients (%)
Resected volume of ACL <25% of ACL volume 25–50% of ACL volume	15 (35.7%) 27 (64.3%)
Concomitant notchplasty Using posterior septal portal	36 (81.8%) 20 (47.6%)

**Table 4:** Preoperative and final follow-up clinical outcomes (*n* = 42).

Parameter	Preoperative	Final Follow-Up	*p*-Values ^b^ (95% CI)
Flexion contracture ^a^	5.12° ± 1.07° (range, 0–30°)	0.6° ± 0.30° (range, 0–10°)	<0.001
Further flexion ^a^	128.21° ± 2.36° (range, 80–140°)	137.62° ± 0.80° (range, 120–140°)	<0.001
Range of motion ^a^	123.1° ± 2.65°	137.02° ± 0.98°	<0.001
Lysholm knee score ^a^	64.62 ± 2.81	87.76 ± 1.94	<0.001
HSS score ^a^	71.74 ± 2.68	94.14 ± 0.99	<0.001

HSS score = Hospital for Special Surgery knee score. CI = Confidence interval. ^a^ Values are given as mean and standard deviation. ^b^ *p*-values obtained from paired *t*-test or Wilcoxon signed-rank test as appropriate.

**Table 5:** Postoperative clinical outcomes between the two groups (*n* = 42).

Parameters	Group 1 (*n* = 15)	Group 2 (*n* = 27)	*p*-Value ^c^ (95%CI)
Flexion contracture ^b^	1.0° ± 2.80° (range, 0–10°)	0.4° ± 1.33° (range, 0–5°)	0.423
Further flexion ^b^	137.3° ± 5.94° (range, 120–140°)	137.7° ± 4.80° (range, 120–140°)	0.795
Lysholm knee score ^b^	136.3 ± 8.12	137.4 ± 5.26	0.606
HSS score ^b^	93.4 ± 6.53	94.56 ± 6.43	0.582
Notchplasty ^a^	10 (66.7%)	26 (96.3%)	0.008

HSS score = Hospital for Special Surgery knee score. CI = Confidence interval. ^a^ Values are given as a number. ^b^ Values are given as mean and standard deviation. ^c^ *p*-values obtained from independent t-test or Mann–Whitney test as appropriate.
